# Genetic characterization of Moroccan and the exotic bread wheat cultivars using functional and random DNA markers linked to the agronomic traits for genomics-assisted improvement

**DOI:** 10.1007/s13205-016-0413-y

**Published:** 2016-04-06

**Authors:** Fatima Henkrar, Jamal El-Haddoury, Hassan Ouabbou, Najib Bendaou, Sripada M. Udupa

**Affiliations:** 1 ICARDA-INRA Cooperative Research Project, International Center for Agricultural Research in the Dry Areas (ICARDA), B.P. 6299, Rabat, Morocco; 2 Institut National de la Recherche Agronomique (INRA), B.P. 415, Rabat, Morocco; 3 Institut National de la Recherche Agronomique (INRA), B.P. 589, Settat, Morocco; 4 Laboratoire de Physiologie et Biotechnologie Végétale, Faculté des Sciences, Université Mohammed V, B.P. 1014, Rabat, Morocco

**Keywords:** Genetic diversity, Functional markers, Linked random DNA, Agronomic traits, Bread wheat

## Abstract

Genetic characterization, diversity analysis and estimate of the genetic relationship among varieties using functional and random DNA markers linked to agronomic traits can provide relevant guidelines in selecting parents and designing new breeding strategies for marker-assisted wheat cultivar improvement. Here, we characterize 20 Moroccan and 19 exotic bread wheat (*Triticum aestivum* L.) cultivars using 47 functional and 7 linked random DNA markers associated with 21 loci of the most important traits for wheat breeding. The functional marker analysis revealed that 35, 45, and 10 % of the Moroccan cultivars, respectively have the rust resistance genes (*Lr34/Yr18/Pm38*), dwarfing genes (*Rht1b* or *Rht2b* alleles) and the leaf rust resistance gene (*Lr68*). The marker alleles for genes *Lr37/Yr17/Sr38*, *Sr24* and *Yr36* were present only in the exotic cultivars and absent in Moroccan cultivars. 25 % of cultivars had *1BL.1RS* translocation. 70 % of the wheat cultivars had *Ppo*-*D1a* and *Ppo*-*A1b* associated with low polyphenol oxidase activity. 10 % of cultivars showed presence of a random DNA marker allele (175 bp) linked to Hessian fly resistance gene *H22*. The majority of the Moroccan cultivars were carrying alleles that impart good bread making quality. Neighbor joining (NJ) and principal coordinate analysis based on the marker data revealed a clear differentiation between elite Moroccan and exotic wheat cultivars. The results of this study are useful for selecting suitable parents for making targeted crosses in marker-assisted wheat breeding and enhancing genetic diversity in the wheat cultivars.

**Electronic supplementary material:**

The online version of this article (doi:10.1007/s13205-016-0413-y) contains supplementary material, which is available to authorized users.

## Introduction

Wheat (*Triticum aestivum* L.) is an important staple crop, providing 20 % of all calories consumed by people worldwide. Demand for wheat is predicted to increase in the future as the global population increases. With the world’s population estimated to reach 9.6 billion by 2050, wheat production will have a crucial bearing on food security and the global economy in the coming decades. In Morocco, wheat is the most consumed cereal crop, with a per capita consumption of 258 kg annually (USDA Foreign Agricultural Service 2014). In Morocco, it is cultivated in an area of 3.2 million ha, mostly in rainfed conditions with a production of 6.9 million tonnes in 2013 (FAOSTAT 2014). Its productivity is comparatively low, due to abiotic stresses such as drought, and biotic stresses such as Hessian fly, leaf rust, and yellow rust. Consequently, Morocco is not self sufficient in wheat production in most of the years and imports bread wheat for its domestic consumption. Therefore, the overall objectives of wheat breeding remains the development of wheat genotypes with higher yield, higher adapted to contrasted environment, resistance to the biotic stresses and with acceptable higher grain quality. Even all the effort made for improving wheat, its productivity still depends on traditional breeding and selection using conventional techniques. Currently, the Moroccan breeding program is giving a priority to new technologies such as the use of molecular markers to speed up the development of improved wheat varieties.

The characterization of genetic variability and an estimate of the genetic relationship among varieties are essential to any breeding program; because of artificial crosses among less similar parents allow a larger segregation and the combination of different favorable alleles (Bered et al. 2002). Genetic similarities might be evaluated by means of pedigree analysis (Barrett and Kidwell 1998) or by assessing morphological traits (Schut et al. 1997) as well as biochemical (Metakovsky and Branlard 1998) or, more recently, DNA markers (Barrett and Kidwell 1998; Pagnotta et al. 2005). The use of molecular approaches, particularly molecular markers, has allowed better characterization, maintenance of genetic variation in plant germplasm, identifying genes underlying important traits, and devising optimal breeding strategies for crop improvement (Hayden et al. 2010). Therefore, evaluation of the genetic diversity present in wheat germplasm deployed in the current breeding programs at the molecular level and integration of this information into cultivar development are essential for using genetic resources effectively in breeding programs (Chao et al. 2007).

Identification of molecular markers that cosegregate or closely linked with the agronomic traits is useful for marker-assisted selection (MAS; Mohan et al. 1997). Closely linked random DNA markers (RDMs; Andersen and Lubberstedt 2003) and gene specific or functional markers (Andersen and Lubberstedt 2003) are commonly used for MAS. In contrast to random DNA markers, gene specific or functional markers are ideal for MAS in wheat breeding as they are derived directly from the gene conferring the phenotype. In plant breeding, functional markers can be used for validation of cultivar identity, selection of parental materials to build segregating population, and subsequent selection of lines (Lübberstedt et al. 2005). Several markers were developed and validated for MAS. To date, more than 30 wheat loci associated with end-use quality, agronomic traits, and disease resistance in bread wheat (Liu et al. 2012). 56 functional markers for quality traits such as high- and low-molecular-weight glutenin subunits (HMW-GS and LMW-GS), polyphenol oxidase (PPO) activity, lipoxynase (LOX) activity, yellow pigment content (YPC), kernel hardness (*Pin*), and starch properties have been developed. 27 functional markers for agronomic traits were developed and reportedly used in wheat breeding programs such as semi-dwarfing genes *Rht*-*B1b* (*Rht1*) and *Rht*-*D1b* (*Rht2*), photoperiod response genes (*Ppd*), vernalization genes (*Vrn*) and developmental rate genes. For rust disease resistance, six genes *Lr34/Yr18/Pm38*, *Lr37/Yr17/Sr38*, *Lr19*, *Lr47*, *Lr51* and *Yr36* had been cloned in wheat (Feuillet et al. 2003; Huang et al. 2003; Yahiaoui et al. 2004; Fu et al. 2009; Krattinger et al. 2009; Liu et al. 2014) in addition to 1B/1R translocation (Froidmont 1998; Chai et al. 2006; Liu et al. 2008) and functional markers were designed and successfully applied in the breeding. The objective of this work was to genotype 20 Moroccan and 19 exotic bread wheat cultivars using 47 functional markers and 7 random DNA markers closely linked to 21 loci of the most important target traits for breeding and to determine the genetic relationship between them to identify the potential parental lines for the wheat breeding programs.

## Materials and methods

### Plant materials

A set of 39 wheat lines, which includes 20 improved elite cultivars of Morocco and 19 exotic cultivars (Table 1) were used for the marker analysis. The exotic wheat lines were introduced to Morocco to be used as donors for the specific traits of interest in the wheat breeding program. The Moroccan cultivars were procured from the National Gene Bank of Morocco, whereas, the exotic cultivars were procured from the national or international gene banks of the other countries.

**Table 1:** Cultivar name and pedigree of Moroccan and the exotic bread wheat used in this study

Cultivar	Origin	Pedigree^a^
Saïs	Morocco	Tob’s’/1/NP/2/CC/Inia/3/Cha
Arrehane	Morocco	L222 introduced from USA
Acsad-59	Morocco	Selection from Arab Center for the studies of arid zones and dry lands (ACSAD) nursery
Kanz	Morocco	Pavon’s’/4/Pato (R)/1/Cal/3/7C/2/Bb/Cno
Aguilal	Morocco	Saïs*2/1/KS-85-14-2
Tilila	Morocco	Veery ‘s’
Achtar	Morocco	Hork/1/Ymh/2/Kal/1/Bb
Nasma	Morocco	Moroccan selection
Khair	Morocco	Maya/2/LR64/1/LR64/3/TZPP/1/Y54/2/23584
Massira	Morocco	L2266/1/1406,101/2/Buc’s’/3/Vpm/1/Mos 83,11,4,8/2/Nac
Mehdia	Morocco	Kauz’S’
Rajae	Morocco	Mor’s’/1/Mon’s’
Amal	Morocco	Bow’s’/1/Buc’s’
Baraka	Morocco	Vent71/2/Cno67’s’/1/SC66/3/Kal/1/Bb (=Pavon)
Jouda	Morocco	Kal/1/blue bird
Saba	Morocco	Nasma/1/PotamPRL/2*PASTOR
Marchouch	Morocco	Kal/1/Ciano/2/8156^2^/3/BT908
Potam	Morocco	Selection from CIMMYT nursery
Saada	Morocco	Butte/2/Arthur/1/Butte
Salama	Morocco	Introduced from Europe by SONACOS, Morocco
Yecora Rojo-Gpc-B1/Yr36	USA	Fa-15-3(Tr.Ds,Isr)/7*Yecora Rojo
Pavon-76	Mexico	Vicam-71//Ciano-67/Siete-Cerros-66/3/Kalyansona/Bluebird
Parula	Mexico	Frontana/Kenya-58//Newthatch/3/2*Frocor//Kenya-Ad/Gabo-54/4/Bluebird/Chanate;Frontana/Kenya-58//Newthatch/3/2*Frontana//Kenya-350/Gabo-55/4/Bluebird/Chanat
Opata-85	Mexico	Bluejay(Sib)/Jupateco-73
Dharwar Dry	India	–
Stylet	Australia	Molineux/2*Trident
Annuello	Australia	Pavon(Sib)/Tm-56(Vf-665)//Janz
Chinese Spring	China	Old accession
Lew	USA	Fortuna, Usa/S-6285
Sumai-3	China	Funo/Taiwan-Xiaomai; Jingzhou/Sumai-2; Funo/Taiwanmai
Bobwhite-S	Mexico	Avrora//Kalyansona/Bluebird/3/(Sib)Woodpecker
Rampart	USA	Lew/Tiber//Redwin
Veranopolis	Brazil	Trintecinco/Frontana
Veery	Mexico	Kavkaz/Buho//Kalyansona/Bluebird
Frontana	Brazil	Fronteira/Mentana
Largo	USA	Langdon (Tr.Dr)/(Tr.Ta)Pi-268210; Langdon (Tr.Dr)/(Tr.Ta)Pi-268219
Experiment Station-88	Bulgaria	Bulgarian-88
Tadinia	USA	Tadorna(W)/Inia-66
Turksikum	Azerbaijan	PI262660

^a^The nomenclature described in Skovmand et al. (1997) was used for writing pedigrees

### DNA extraction and marker genotyping

Total genomic DNA was extracted by CTAB method of Saghai-Maroof et al. (1984) with some modifications as adapted by Udupa et al. (1999). Fresh young leaves were collected from green house grown plants of individual cultivars. The isolated DNA was estimated both qualitatively and quantitatively using 1.0 % (w/v) agarose gels by comparing bands to known concentrations of lambda DNA.

Total of 47 functional markers and 7 linked random DNA markers (RDMs) to the traits of interest were used for genotyping the bread wheat cultivars. They were *Lr34* (Lagudah et al. 2006), *Lr68* (Herrera-Foessel et al. 2012), *Lr37* (Helguera et al. 2003), *Sr24* (Mago et al. 2005), *Gpc*-*B1/Yr36* (Distelfeld et al. 2006), *Rht*-*B1*, *Rht*-*D1* (Ellis et al. 2002), *Vp1*-*B3* (Yang et al. 2007), *Ppo*-*A1*, *Ppo*-*D1* (He et al. 2007), *Ppd*-*D1* (Beales et al. 2007; Yang et al. 2009), *iag95* for 1B/1R (Mago et al. 2002), *Pina*-*D1* (Gautier et al. 1994), *Allwaxy* (McLauchlan et al. 2001), *Glu*-*A1* (Lafiandra et al. 1997; De Bustos et al. 2000), *Glu*-*B1* (Ahmad 2000; Butow et al. 2004; Lei et al. 2006), *Glu*-*D1* (Ahmad 2000), *Glu*-*A3* (Zhang et al. 2004), *Glu*-*B3* (Wang et al. 2009), *Glu*-*D3* (Zhao et al. 2007) and *Xgdm33*, closely linked to gene *H22* (Zhao et al. 2006). PCR reaction was performed in a reaction volume of 10 μL containing 1× PCR buffer (1.5 mM MgCl_2_), 200 µM of each dNTPs, 10 pmol of each primer, 0.5U of *Taq* DNA polymerase (Promega) and approximately 50 ng of genomic DNA. Primers names, sequences and cycling conditions for each molecular marker are detailed in supplementary Table S1. The PCR products were separated 1.2 or 1.5 % (w/v) agarose gels. Except for *Allwaxy*, *Rht*-*B1*, *Rht*-*D1* and *Xgdm33* were run in 6 % native polyacrylamide gels, prepared in a vertical electrophoresis unit (CBS Scientific) using 0.5 × TBE buffer. The different gels were stained with ethidium bromide and visualized under UV light.

### Analysis of molecular data

PowerMarker software version 3.25 (Liu and Muse 2005) was used to calculate the number of alleles and values of genetic diversity and PIC (Botstein et al. 1980) of each locus. Genetic distances between each pair of cultivars were measured by calculating the shared allele frequencies (Jin and Chakraborty 1993). The Neighbor joining dendrogram was generated using the DARwin software based on the genetic distance calculated using PowerMarker software. The genetic structure was analyzed by performing PCoA (Principal Coordinates analysis) implemented in the program GenAlex 6.5 (Peakall and Smouse 2012).

## Results

### Genetic diversity analysis

Genetic diversity of 20 elite Moroccan cultivars and 19 potential exotic cultivars to be deployed in the breeding program was evaluated using 47 functional and 7 random DNA markers linked to the target traits of interest. The total number of detected alleles was 48 in Moroccan cultivars and 56 in exotic cultivars. Average number of alleles was slightly higher in exotic cultivars than Moroccan cultivars. Mean number of alleles was 2.5 and 2.9 in Moroccan and exotic cultivars, respectively. Similarly, exotic cultivars had a higher PIC value (0.39) compared to Moroccan cultivars (0.34) (Table 2). The 54 primer pairs for specific alleles linked to 21 loci distributed in 12 chromosomes showed a good polymorphism in Moroccan cultivars with slight difference to the exotic cultivars. The genetic diversity calculated was 0.4. The glutenin genes namely *Glu*-*B1*, *Glu*-*A3* and *Glu*-*B3* were the most polymorphic and displayed higher number of alleles (5, 5 and 6) and high genetic diversity (0.735, 0.660 and 0.770), respectively.

**Table 2:** Major allele frequency, number of alleles, genetic diversity and PIC at functional and random DNA markers linked to end-use quality, agronomic traits, and biotic stresses resistance in Moroccan and the exotic bread wheat cultivars

Marker	Chromosome	Moroccan cultivars				Exotic cultivars			
		Sample size	No. of alleles	Gene diversity	PIC	Sample size	No. of alleles	Gene diversity	PIC
*Lr34/Yr18/Pm38*	7D	20	2	0.455	0.351	19	2	0.487	0.368
*Rht*-*B1 (Rht1) *	4B	20	2	0.495	0.372	19	2	0.432	0.400
*Rht*-*D1 (Rht2)*	4D	20	2	0.495	0.372	19	2	0.265	0.231
*iag95*	1B/1R	20	2	0.420	0.332	19	2	0.188	0.170
*Pina*- *D1*	5D	20	2	0.375	0.305	19	2	0.487	0.368
*Wx*-*A1*	1A	20	2	0.420	0.332	19	2	0.100	0.094
*Wx*-*B1*	4A	20	2	0.375	0.305	19	2	0.332	0.277
*Ppd*-*D1*	2D	20	2	0.255	0.222	19	2	0.487	0.368
*Vp1*-*B3*	3B	20	1	0.000	0.000	19	3	0.460	0.392
*Lr68*	7B	20	2	0.180	0.164	19	2	0.188	0.171
*Ppo*-*D1*	2D	20	2	0.420	0.332	19	2	0.432	0.338
*Ppo*-*A1*	2A	20	2	0.420	0.332	19	2	0.387	0.312
*Xgdm33*-*H22*	1D	20	2	0.255	0.222	19	2	0.332	0.277
*Glu*-*A1*	1A	20	3	0.555	0.491	15	3	0.638	0.561
*Glu*-*B1*	1B	20	5	0.735	0.690	19	5	0.714	0.664
*Glu*-*D1*	1D	20	2	0.255	0.222	15	3	0.560	0.461
*Glu*-*A3*	1A	20	5	0.660	0.611	19	7	0.800	0.770
*Glu*-*B3*	1B	17	6	0.770	0.736	17	8	0.844	0.825
*Glu*-*D3*	1D	20	2	0.095	0.090	15	3	0.417	0.369
Total			48				56		
Mean			2.526	0.402	0.341		2.947	0.450	0.387
SD (±)			1.307	0.201	0.187		1.779	0.200	0.195

### Markers based trait analysis

The 20 elite Moroccan cultivars were screened with the functional and the random DNA markers linked with quality, agronomic traits, and disease resistance (Table 3). The frequency of leaf rust resistance functional allele at *Lr34* gene was 35 % and at *Lr68* gene the linked random DNA marker allele was 10 % (Table 3). The marker alleles for genes *Lr37*, *Sr24* and *Yr36* were absent in Moroccan cultivars, whereas they were present only in exotic cultivars. 25 % of cultivars had 1R segment (1BL.1RS translocation) and 10 % of cultivars showed presence of 175 bp size allele of *Xgdm33* linked with Hessian fly resistance gene *H22*. For the other agronomic traits, such as, dwarfing genes *Rht1* and *Rht2*, the frequency was 45 % for each gene. Majority of wheat cultivars had *Ppo*-*D1a* and *Ppo*-*A1b* alleles associated with low polyphenol oxidase activity (70 %). Only 15 % of the cultivars had photoperiod insensitive allele at *Ppd*-*D1* locus. While, *Vp1*-*B3* STS primer pair amplified 569 bp fragment linked to preharvest sprouting tolerance in all 20 Moroccan cultivars. For the end-use quality traits, the frequency of *wx*-*A1* and *wx*-*B1* associated to improved starch quality was 70 and 75 %, respectively. In addition, *wx*-*D1* (data not shown) existed in all Moroccan and exotic cultivars. Twenty-five percent of cultivars carried *Pina*-*D1a* linked to soft grain texture. The Glutenin genes revealed high level of polymorphism related to variable degree of bread making quality.

**Table 3:** Moroccan and exotic cultivars showing presence of important genes/traits of interest to wheat breeding based on analysis of the functional and random DNA markers linked to the agronomic traits

Locus	Type of marker	Interesting allele designation/size in bp	Allele frequency (%)	Cultivars
*Lr34/Yr18/Pm38*	Functional	150 bp	35	Moroccan cultivars: Arrehane, Acsad-59, Massira, Mehdia, Baraka, Jouda and Saada
			42	Exotic cultivars: Parula, Opata-85, Dharwar Dry, Annuello, Chinese Spring, Sumai-3, Veranopolis and Frontana
*Rht*-*B1* (*Rht1*)	Functional	*Rht*-*B1b* (237 bp)	45	Moroccan cultivars: Arrehane, Acsad-59, Tilila, Achtar, Khair, Massira, Mehdia, Baraka and Jouda
			37	Exotic cultivars: Yecora Rojo-Gpc-B1/Yr36, Dharwar Dry, Opata-85, Annuello, Bobwhite-S, Veery and Tadinia
*Rht*-*D1* (*Rht2*)	Functional	*Rht*-*D1b* (254 bp)	45	Moroccan cultivars: Saïs, Kanz, Aguilal, Nasma, Rajae, Amal, Marchouch, Potam and Salama
			16	Exotic cultivars: Yecora Rojo-Gpc-B1/Yr36, Pavon-76 and Stylet
*iag95* (1BL/1RS)	Closely linked	1.1 kb	25	Moroccan cultivars: Tilila, Mehdia, Rajae, Amal and Salama
			11	Exotic cultivars: Bobwhite-S and Veery
*Pina*-*D1* (Softness)	Functional	*Pina*-*D1a* (330 bp)	25	Moroccan cultivars: Saïs, Acsad-59, Aguilal, Massira and Potam
			58	Exotic cultivars: Stylet, Annuello, Chinese Spring, Lew, Sumai-3, Rampart, Veranopolis, Frontana, Largo, Experiment Station-88 and Turksikum
*Wx*-*A1*	Functional	257 bp	70	Moroccan cultivars: Saïs, Arrehane, Aguilal, Tilila, Nasma, Khair, Massira, Amal, Baraka, Saba, Marchouch, Potam, Saada and Salama
			95	Exotic cultivars: Yecora Rojo-Gpc-B1/Yr36, Parula, Opata-85, Dharwar Dry, Stylet, Annello, Chinese Spring, Lew, Sumai-3, Bobwhite-S, Rampart, Veranopolis, Veery, Frontana, Largo, Experiment Station-88, Tadinia and Turksikum
*Wx*-*B1*	Functional	227 bp	75	Moroccan cultivars: Saïs, Acsad-59, Kanz, Aguilal, Tilila, Nasma, Khair, Mehdia, Rajae, Amal, Saba, Marchouch, Potam, Saada and Salama
			79	Exotic cultivars: Yecora Rojo-Gpc-B1/Yr36, Parula, Opata-85, Dharwar Dry, Chinese Spring, Lew, Sumai-3, Bobwhite-S, Rampart, Veranopolis, Veery, Largo, Experiment Station-88, Tadinia and Turksikum
*Ppd*-*D1*	Functional	*Ppd*-*D1a* (414 bp)	15	Moroccan cultivars: Nasma, Saba and Saada
			42	Exotic cultivars: Dharwar Dry, Stylet, Chinese Spring, Lew, Rampart, Largo, Experiment Station-88 and Turksikum
*Vp1*-*B3*	Functional	569 bp	100	Moroccan cultivars: All cultivars studied
			74	Exotic cultivars: Yecora Rojo-Gpc-B1/Yr36, Pavon-76, Parula, Opata-85, Dharwar Dry, Stylet, Annuello, Chinese Spring, Lew, Sumai-3, Bobwhite-S, Rampart, Veery and Largo
*Lr68*	Closely linked	385 bp	10	Moroccan cultivars: Saada and Salama
			10	Exotic cultivars: Parula and Frontana
*Ppo*-*D1*	Functional	*Ppo*-*D1a* (730 bp for PPO16 and Null for PPO29)	70	Moroccan cultivars: Arrehane, Acsad-59, Kanz, Tilila, Achtar, Nasma, Khair, Massira, Rajae, Amal, Baraka, Marchouch, Saada, Salama
			68	Exotic cultivars: Pavon-76, Parula, Opata-85, Dharwar Dry, Annuello, Chinese Spring, Sumai-3, Bobwhite-S, Veranopolis, Frontana, Largo, Experiment Station-88 and Turksikum
*Ppo*-*A1*	Functional	*Ppo*-*A1b* (685 bp for PPO18 and 290 bp for PPO33)	70	Moroccan cultivars: Achtar, Nasma, Massira, Rajae, Amal and Marchouch
			26	Exotic cultivars: Opata-85, Chinese Spring, Sumai-3, Bobwhite-S and Veery
*H22*	Linked	175 bp	10	Moroccan cultivars: Arrehane and Aguilal
			0	Exotic cultivars: Nill

### Genetic relationships and PCoA analysis

**Fig. 1:**
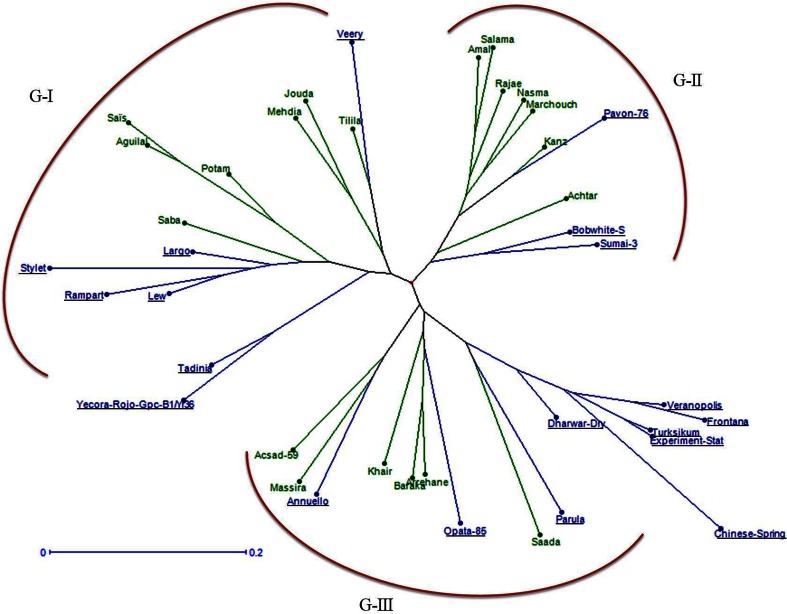
The Neighbor joining dendrogram generated based on shared allele genetic distance among 20 Moroccan cultivars and 19 exotic cultivars (*names underlined*) of bread wheat. All the cultivars were clustered into three major groups (G-I, G-II and G-III)

To study the genetic relationships between Moroccan and exotic cultivars for breeding purposes, the allelic data were used to estimate the genetic distance between all cultivars and Neighbor joining dendrogram was generated (Fig. 1). All cultivars were clustered into three major groups (G-I, G-II and G-III). In each group, the Moroccan and exotic cultivars were mainly separated into subgroups. However, Mexican cultivars Pavon-76, Veery and Opata-85 and an Australian cultivar Annuello were grouped with Moroccan cultivars. The most divergent pair was Chinese Spring and Moroccan cultivars Jouda, Mehdia and Saïs, which exhibited highest genetic distance (0.74). The two Moroccan cultivars Aguilal and Saïs were genetically close (0.11). Similarly, genetic distance between exotic cultivars Frontana and Veranopolis were smallest (0.11).

**Fig. 2:**
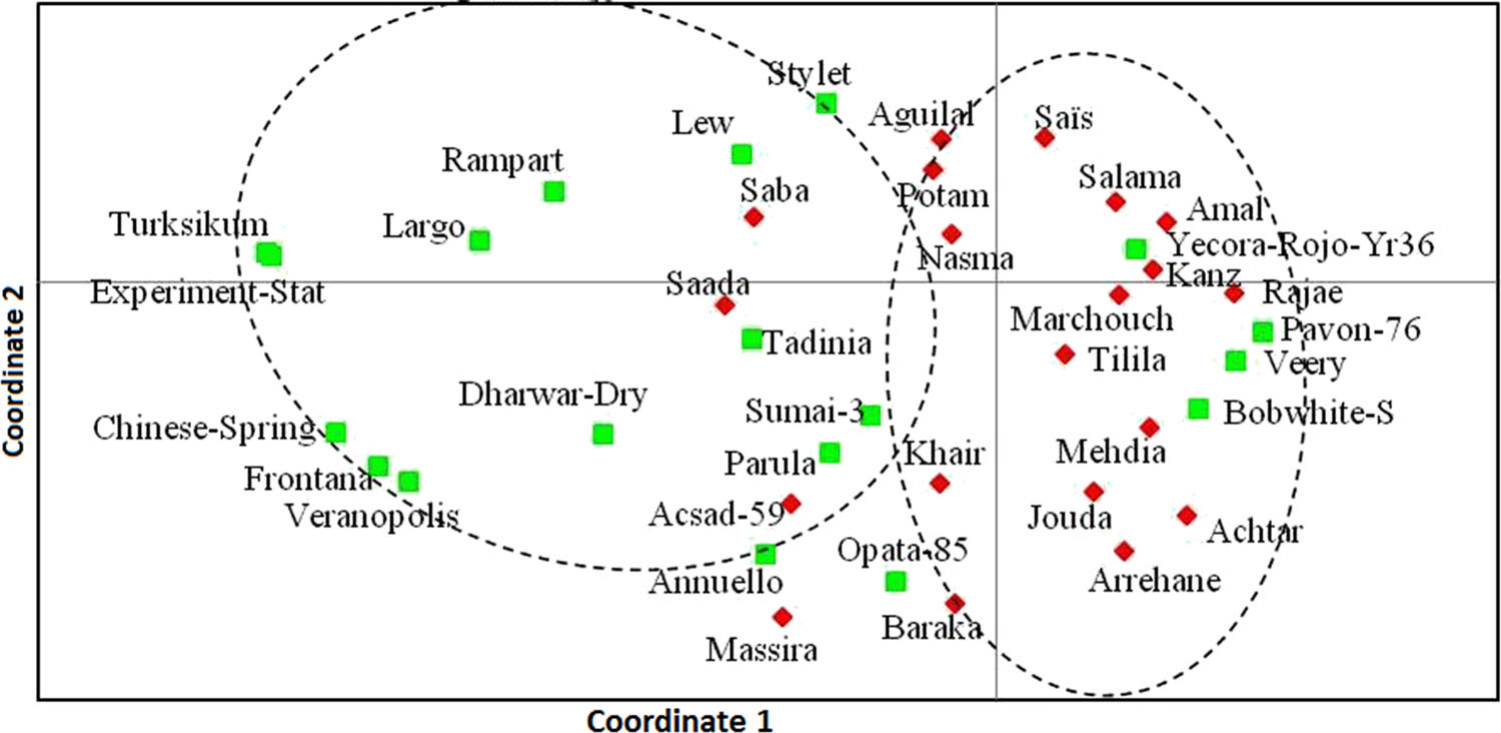
Principal coordinate analysis (PCoA) plot generated from genetic distance calculations using the GENALEX package for 20 Moroccan cultivars (marked in *square*) and 19 exotic cultivars (marked in *diamond*) of bread wheat

The genetic structure was analyzed using principal coordinates analysis (PCoA). The PCoA of genetic distance between genotypes, based on gene frequencies revealed differentiation between cultivars. The three axes explained 16.38, 13.39 and 9.61 % of the total variance, and separated the cultivars into two clusters, Moroccan cultivars in one cluster and exotic cultivars in another cluster (Fig. 2), except, the exotic Mexican cultivars Pavon-76, Veery and Bobwhite and the American cultivars Yecora Rojo-Gpc-B1/Yr36 were grouped with the Moroccan cluster.

## Discussion

The Moroccan wheat cultivars used in this study represent the most advanced breeding lines released for cultivation in Morocco and encompass important gene pools adapted to Morocco and the North Africa region. Therefore, information of genetic diversity, identification of specific alleles, genes or loci and assessment of the genetic relationships among these cultivars can provide relevant guidelines in selecting parents and for designing new breeding strategies for wheat cultivar improvement, especially, against leaf rust, yellow rust and Hessian fly, which are considered as most destructive biotic stresses in Morocco (Elhaddoury et al. 2012). Lombardi et al. (2014) reported that selection of divergent parental genotypes for breeding should be made actively on the basis of systematic assessment of genetic distance between genotypes, rather than passively based on geographical distance.

The total number of alleles detected at 21 loci was 48 alleles in Moroccan cultivars (mean 2.5 alleles) and 56 alleles in the exotic cultivars (mean 2.9 alleles). The PIC value was 0.34 for Moroccan cultivars and 0.39 for exotic cultivars. Similar studies have been conducted by Vanzetti et al. (2013) for 102 Argentinean bread wheat cultivars and reported an average number of alleles and PIC values of 3.26 and 0.458, respectively. In India, Malik et al. (2013) characterized 48 elite Indian wheat genotypes and reported 2.42 alleles per locus and 0.4596 PIC value.

The functional markers and the random DNA markers linked to the target traits such as the rust resistance (*Lr34*, *Lr68, Lr37*, *Yr36* and *Sr24*), Hessian fly resistance (*H22*), 1BL/1RS translocation, growth photoperiod sensitivity (*Ppd*-*D1*), plant height (*Rht*-*B1*, *Rht*-*D1*), grain texture (*Pina*-*D1*), starch waxy proteins variants (*Wx*-*A1*, *Wx*-*B1*), PPO activity (*Ppo*-*A1*, *Ppo*-*D1*), pre-harvest sprouting tolerance (*Vp1*-*B3*), high molecular weight glutenins (*Glu*-*A1*, *Glu*-*B1*, *Glu*-*D1*) and low molecular weight glutenins (*Glu*-*A3*, *Glu*-*B3*, *Glu*-*D3*) shown to be ideal for marker-assisted selection in wheat breeding. The information generated in this study is also useful for selection of parental materials to develop segregating population for marker-assisted selection. The use of gene specific markers permitted to know the genetic structure of Moroccan modern wheat cultivars. The functional alleles of some of these traits were very well related to the respective phenotypes of the cultivars, previously described by the breeders. For instance, the cultivars Arrehane and Aguilal known for their resistance to Hessian fly (Jlibene and Nsarellah 2011), and carrying the *H22* gene (Lhaloui et al. 2000), were clearly amplified the allele of the marker *Xgdm33* tightly linked to *H22* (Zhao et al. 2006). In addition, Arrehane showed the presence of durable resistance gene *Lr34/Yr18/Pm38* and dwarfing gene allele *Rht-B1b*. Other cultivars namely Baraka, Acsad-59, Jouda and Mehdia which were positive for *Lr34/Yr18/Pm38* and *Rht-B1b* dwarfing gene allele are also known for their large adaptation, high yield and tolerance to drought (Jlibene and Nsarellah 2011). However, these cultivars need to be further improved by incorporating the Hessian fly resistance, which is very important problem in arid and semi-arid regions of Morocco and the North Africa. Based on the marker analysis, the cultivars Saada and Massira with resistance to the Hessian fly (*H5* gene; Lhaloui et al. 2000) were also found to be carrying *Lr34*/*Yr18/Pm38* slow rusting gene. The linked random DNA analysis also revealed the possibilities of having the second slow rusting gene *Lr68* in Saada and Massira, which needs to be further confirmed based on the phenotypic characterization. These two cultivars with two slow rust resistance genes could be a valuable parent in wheat breeding program due the additive resistance effect resulted from combined slow rusting genes (Lillemo et al. 2011). Furthermore, the analysis in this study also revealed that the cultivar Saada also carried photoperiod insensitive allele *Ppd*-*D1a* (Yang et al. 2009) and waxy locus allele *wx*-*B1* associated with improved starch quality (McLauchlan et al. 2001). Therefore, Saada is very valuable cultivar for use as donors in molecular breeding program. The cultivars Tilila and Mehdia revealed the presence of *iag95* marker specific for 1BL.1RS translocation. Tilila showed also presence of waxy allele *wx*-*A1* and *wx*-*B1*, low polyphenol oxidase activity alleles (*Ppo*-*D1a* and *Ppo*-*A1b*). This cultivar (Tilila) is known in Morocco for its large adaptation, moderate yield and resistance against many diseases (Jlibene 1996).

Estimation of the degree of differentiation between cultivars that are included in a crossing program is useful for selection of parental genotypes. The Mexican cultivars Pavon-76, Bobwhite and Veery were genetically closer to Moroccan cultivars. Based on the knowledge of pedigrees of exotic and Moroccan cultivars and the history of Moroccan breeding, it is known that Mexican cultivars and CIMMYT germplasm were extensively used in Morocco since 1980s (Jlibene and Nsarellah 2011). Most of the Moroccan cultivars had Pavon’s and Veery or their common parents such as Bluebirds and Kalyansona as parents (Skovmand et al. 1997). The NJ dendrogram and PCoA results revealed a clear differentiation between Moroccan and the exotic cultivars deployed in the current breeding program indicating that the exotic cultivars used in this study, were divergent from Moroccan cultivars and can be used to improve disease resistance, quality and also genetic diversity.

## Electronic supplementary material

Below is the link to the electronic supplementary material.

Supplementary material 1 (DOCX 28 kb)
