# Supplementary material for: Genetic characterization of Moroccan and the exotic bread wheat cultivars using functional and random DNA markers linked to the agronomic traits for genomics-assisted improvement
Source: 3 Biotech. 2016 Apr 6;6(1):97. doi: 10.1007/s13205-016-0413-y (PMC4823230; doi:10.1007/s13205-016-0413-y)
Supplement: Supplementary file 1 — Supplementary material 1 (DOCX 28 kb) [file 13205_2016_413_MOESM1_ESM.docx]

**Table S1**: Locus name, primers names, sequences and PCR cycling conditions for each of the molecular marker used in this study

| **Locus name** | **Primer name** | **Forward primer (5’-3’)** | **Reverse primer (5’-3’)** | **PCR cycling conditions** | **Reference** |
| --- | --- | --- | --- | --- | --- |
| *Lr34* | *csLV34* | GTTGGTTAAGACTGGTGATGG | TGCTTGCTATTGCTGAATAGT | 94°C, 5 min  40 cycles of:  [94°C, 45 s; 55°C, 30 s; 72°C, 60 s]  72°C, 7 min | Lagudah et al., 2006  http://maswheat.ucdavis.edu/protocols/Lr34/index.htm |
| *Lr68* | *csGS* | AAGATTGTTCACAGATCCATGTCA | GAGTATTCCGGCTCAAAAAGG | 93°C, 1 min  30 cycles of: [ 93°C, 30 s ; 60°C, 1 min; 72°C, 1 min]  72°C, 5 min | Herrera-Foessel et al., 2012  http://maswheat.ucdavis.edu/protocols/Lr68/index.htm |
| *Lr37* | Ventriup/Ln2 | AGG GGC TAC TGA CCA AGG CT | TGC AGC TAC AGC AGT ATG TAC ACA AAA | 94°C, 5min  30 cycles of: [94°C, 45 s ; 65°C, 30 s ; 72°C, 60 s]  72°C, 7 min | Helguera et al., 2003  http://maswheat.ucdavis.edu/protocols/Lr37/index.htm |
| *Sr24* | Sr24#50 | CCCAGCATCGGTGAAAGAA | ATGCGGAGCCTTCACATTTT | 20°C, 1 min  94°C, 3 min  30 cycles of: [94°C, 30 s; 63°C, 30 s; 72°C, 40 s]  72°C, 10 min | Mago et al., 2005  http://maswheat.ucdavis.edu/protocols/Sr24/index.htm |
| *Yr36* | *UHW89* | TCTCCAAGAGGGGAGAGACA | TTCCTCTACCCATGAATCTAGCA | 94°C , 5 min  37 cycles of: [94°C, 30s; 59°C, 30s; 72°C, 45s]  72°C, 5 min | Ellis et al., 2002  http://maswheat.ucdavis.edu/protocols/Dwarf/index.htm |
| *Rht-B1a* | BF/WR1 | GGT AGG GAG GCG AGA GGC GAG | CAT CCC CAT GGC CAT CTC GAG CTG | 94°C, 5 min  7 cycles of: [94°C, 30 s; First cycle 65°C, 60 s. 1°C drop at every next cycle; 72°C, 80 s]  30 cycles of : [94°C, 15 s ; 58°C, 15 s; 72°C, 50 s]  72°C, 2 min | Ellis et al., 2002  http://maswheat.ucdavis.edu/protocols/Dwarf/index.htm |
| *Rht-B1b* | BF/MR1 | GGT AGG GAG GCG AGA GGC GAG | CAT CCC CAT GGC CAT CTC GAG CTA | 94°C, 5 min  7 cycles of: [94°C, 30 s; First cycle 65°C, 60 s. 1°C drop at every next cycle; 72°C, 80 s]  30 cycles of : [94°C, 15 s ; 58°C, 15 s; 72°C, 50 s]  72°C, 2 min | Ellis et al., 2002  http://maswheat.ucdavis.edu/protocols/Dwarf/index.htm |
| *Rht-D1a* | DF2/WR2 | GGC AAG CAA AAG CTT CGC G | GGC CAT CTC GAG CTG CAC | 95°C, 5 min  30 cycles of: [94°C, 20 s; 58°C, 30 s; 72°C, 10 sec  72°C, 2 min | Ellis et al., 2002  http://maswheat.ucdavis.edu/protocols/Dwarf/index.htm |
| *Rht-D1b* | FD/MR2 | CGC GCA ATT ATT GGC CAG AGA TAG | CCC CAT GGC CAT CTC GAG CTG CTA | 94°C, 5 min  7 cycles of: [94°C, 30 s; First cycle 65°C, 60 s. 1°C drop at every next cycle; 72°C, 80 s]  30 cycles of : [94°C, 15 s ; 58°C, 15 s; 72°C, 50 s]  72°C, 2 min | Ellis et al., 2002  http://maswheat.ucdavis.edu/protocols/Dwarf/index.htm |
| *Vp1-B3* |  | TGCTCCTTTCCCAATTGG | ACCCTCCTGCAGCTCATT G | 94°C, 5 min  36 cycles of: [94°C, 1 min; 61°C, 1 min; 72°C, 1 min]  72°C, 10 min | Yang et al., 2007  http://maswheat.ucdavis.edu/protocols/PHS/index.htm |
| *Ppo-D1a* | *PPO16* | TGCTGACCGACCTTGACTCC | CTCGTCACCGTCACCCGTAT | 94°C, 4 min  30 cycles of: [ 94°C, 1 min; 64°C, 1min; 72°C, 1 min] 72°C, 10 min | He et al., 2007 |
| *Ppo-D1b* | *PPO29* | TGAAGCTGCCGGTCATCTAC | AAGTTGCCCATGTCCTCGCC | 94°C, 4 min  30 cycles of: [ 94°C, 1 min; 64°C, 1 min; 72°C, 1 min] 72°C, 10 min | He et al., 2007 |
| *Ppo-A1a* and *Ppo-A1b* | *PPO18* | AACTGCTGGCTCTTCTTCCCA | AAGAAGTTGCCCATGTCCGC | 94°C, 4 min  30 cycles of: [ 94°C, 1 min; 64°C, 1 min; 72°C, 1 min] 72°C, 10 min | He et al., 2007 |
| *Ppo-A1a and Ppo-A1b* | *PPO33* | CCAGATACACAACTGCTGGC | TGATCTTGAGGTTCTCGTCG | 94°C, 4 min  30 cycles of: [ 94°C, 1 min; 64°C, 1 min; 72°C, 1 min] 72°C, 10 min | He et al., 2007 |
| *Ppd-D1a* |  | ACGCCTCCCACTACACTG | CACTGGTGGTAGCTGAGATT | 94°C, 2 min  40 cycles of: [94°C, 30 s; 52°C, 30 s;  72°C, 1 min]  72°C, 5 min | Yang et al., 2009 |
| *Ppd-D1b* |  | ACGCCTCCCACTACACTG | TGTTGGTTCAAACAGAGAGC | 94°C, 2 min  40 cycles of: [94°C, 30 s; 52°C, 30 s;  72°C, 1 min]  72°C, 5 min | Yang et al., 2009 |
| 1RS/1BL | *iag95* | CTCTGTGGATAGTTACTTGATCGA | CCTAGAACATGCATGGCTGTTACA | 95 °C, 3 min  30 cycles of: [95 °C, 30 s; 50 °C, 50 s; 72°C, 70 s]  72 °C, 10 min | Mago et al., 2002 |
| *Pina-D1a* |  | CCC TGT AGA GAC AAA GCT AA | TCA CCA GTA ATA GCC AAT AGT G | 94°C, 3 min  37 cycles of: [94°C, 90 s ; 55°C, 90 s; 72°C, 2 min]  72°C 10 min | Gautier et al., 1994  http://maswheat.ucdavis.edu/protocols/Hardness/index.htm |
| *Wx-A1, Wx-B1* and *Wx-D1* | *Allwaxy* | AAG AGC AAC TAC CAG T | TCG TAC CCG TCG ATG AAG TCG A | 95ºC, 3 min  2cycles of: [94ºC, 1min; 64ºC-58ºC (1ºC/), 1 min; 72ºC, 1 min]  35 cycles of: [94ºC, 1 min, 58ºC, 1 min; 72ºC, 30 s]  72ºC, 5 min | McLauchlan et al., 2001  http://maswheat.ucdavis.edu/protocols/Waxy/index.htm |
| *Glu-A1* | *Axnull* | ACGTTCCCCTACAGGTACTA | TATCACTGGCTAGCCGACAA | 95°C, 5 min  35 cycles of : [94°C, 1 min; 60°C, 45 s; 72°C, 2 min 10 s]  72°C, 10 min | Lafiandra et al. 1997 |
|  | *Ax1+Ax2** | CCATCGAAATGGCTAAGCGG | GTCCAGAAGTTGGGAAGTGC | 95°C, 5 min  35 cycles of : [94°C, 1 min; 60°C, 45 s; 72°C, 2 min 10 s]  72°C, 10 min | Lafiandra et al. 1997 |
|  | *Ax2** | CCGATTTTGTTCTTCTCACAC | CACCAAGCGAGCTGCAGAT | 95°C, 5 min  35 cycles of : [94°C, 1 min; 57°C, 1 min; 72°C, 2 min 30 s]  72°C, 10 min | De Bustos et al. 2000 |
| *Glu-B1* | *Bx7* | ATGGCTAAGCGCCTGGTCCT | TGCCTGGTCGACAATGCGTCGCTG | 94°C, 5 min  45 cycles of: [94°C, 1 min; 60°C, 1 min; 72°C, 3 min]  72°C for 10 min | Ahmad, 2000 |
|  | *Bx6*, *Bx7* and *Bx7** | CAAGGGCAACCAGGGTAC | AGAGTTCTATCACTGCCTGGT | 94°C, 5min  35 cycles: [94°C, 45 s; 58°C, 45 s; 72°C, 1 min]  72°C, 10 min | Butow et al., 2004; Salmanowicz & Dylewicz, 2007 |
|  | *By18**, *By20**, *By8*, *By8** and *By9* | GCAGTACCCAGCTTCTCAA | CCTTGTCTTGTTTGTTGCC | 94°C, 5min  35 cycles: [94°C, 1 min; 59°C, 1min ; 72°C, 2 min 30 s]  72°C, 10 min | Lei et al., 2006; Salmanowicz & Dylewicz, 2007 |
|  | *By20**,  *By8*, *By8**, *By18** and *By9* | TTCTCTGCATCAGTCAGGA | AGAGAAGCTGTGTAATGCC | 94°C, 5min  35 cycles: [94°C, 1 min; 59°C, 1min ; 72°C, 2 min]  72°C, 10 min | Lei et al., 2006; Salmanowicz & Dylewicz, 2007 |
|  | *By8* | TTAGCGCTAAGTGCCGTCT | TTGTCCTATTTGCTGCCCTT | 94°C, 5min  35 cycles: [94°C, 1 min; 64°C, 1min ; 72°C, 2 min 30 s]  72°C, 10 min | Lei et al., 2006; Salmanowicz & Dylewicz, 2007 |
| *Glu-D1* | *Dx2*, *Dx5* | GCCTAGCAACCTTCACAATC | GAAACCTGCTGCGGACAAG | 94°C, 5 min  45 cycles of: [94°C, 1 min; 63°C; 1 min; 72°C, 1 min]  72°C for 10 min | Ahmad, 2000 |
|  | *Dy10*, *Dy12* | GTTGGCCGGTCGGCTGCCATG | TGGAGAAGTTGGATAGTACC | 94°C, 5 min  45 cycles of: [94°C, 1 min; 63°C; 1 min; 72°C, 1 min]  72°C for 10 min | Ahmad, 2000 |
| *Glu-A3* | *Glu-A3a* | GTACGCTTTTGTAGCTTGTGC | TGGTGGTTGTTGTTGTTGCTACA | 95°C, 3 min  40 cycles of: [ 95°C, 30 s; 59°C, 30 s; 72°C, 1 min] | Zhang et al., 2004 |
|  | *Glu-A3abc* | CACAATTTTCACAGCAACAGCAG | GGCACATTGACACTACACATTG | 95°C, 3 min  40 cycles of: [ 95°C, 30 s; 59°C, 30 s; 72°C, 1 min] | Zhang et al., 2004 |
|  | *Glu-A3ac* | CACAATTTTCACAGCAACAGCAG | TTGGTGGCTGTTGTGAAGACGA | 95°C, 3 min  40 cycles of: [ 95°C, 30 s; 59°C, 30 s; 72°C, 1 min] | Zhang et al., 2004 |
|  | *Glu-A3d* | ACCAGTTATTCATCCATCTGCTC | GTGGTTTCGTACAACGGCTCG | 95°C, 3 min  40 cycles of: [ 95°C, 30 s; 59°C, 30 s; 72°C, 1 min] | Zhang et al., 2004 |
|  | *Glu-A3e* | CAATGAAAACCTTCCTCGTCTG | GATGCCAACGCCTAATGGCACAC | 95°C, 3 min  40 cycles of: [ 95°C, 30 s; 59°C, 30 s; 72°C, 1 min] | Zhang et al., 2004 |
|  | *Glu-A3f* | GTACGCTTTTGTAGCTTGTGC | GTTGCTGCTACAACTGCTGTA | 95°C, 3 min  40 cycles of: [ 95°C, 30 s; 59°C, 30 s; 72°C, 1 min] | Zhang et al., 2004 |
|  | *Glu-A3g* | CAGCAGCCACCACATTCGCAA | GATGCCAACGCCTAATGGCACAC | 95°C, 3 min  40 cycles of: [ 95°C, 30 s; 59°C, 30 s; 72°C, 1 min] | Zhang et al., 2004 |
| *Glu-B3* | *Glu-B3a* | CACAAGCATCAAAACCAAGA | TGGCACACTAGTGGTGGTC | 94°C, 5 min  38 cycles of: [94°C, 35 s; 55°C, 35 s; 72°C, 90 s]  72°C, 8 min | Wang et al., 2009 |
|  | *Glu-B3b* | ATCAGGTGTAAAAGTGATAG | TGCTACATCGACATATCCA | 94°C, 5 min  38 cycles of:[ 94°C, 35 s; 56°C, 35 s; 72°C, 90 s]  72°C, 8 min | Wang et al., 2009 |
|  | *Glu-B3c* | CAAATGTTGCAGCAGAGA | CATATCCATCGACTAAACAAA | 94°C, 5 min  38 cycles of:[ 94°C, 35 s; 56°C, 35 s; 72°C, 90 s]  72°C, 8 min | Wang et al., 2009 |
|  | *Glu-B3d* | CACCATGAAGACCTTCCTCA | GTTGTTGCAGTAGAACTGGA | 94°C, 5 min  38 cycles of:[ 94°C, 35 s; 58°C, 35 s; 72°C, 90 s]  72°C, 8 min | Wang et al., 2009 |
|  | *Glu-B3e* | GACCTTCCTCATCTTCGCA | GCAAGACTTTGTGGCATT | 94°C, 5 min  38 cycles of:[ 94°C, 35 s; 58°C, 50 s; 72°C, 90 s]  72°C, 8 min | Wang et al., 2009 |
|  | *Glu-B3fg* | TATAGCTAGTGCAACCTACCAT | CAACTACTCTGCCACAACG | 94°C, 5 min  38 cycles of:[ 94°C, 35 s; 62°C, 35 s; 72°C, 90 s]  72°C, 8 min | Wang et al., 2009 |
|  | *Glu-B3g* | CCAAGAAATACTAGTTAACACTAGTC | GTTGGGGTTGGGAAACA | 94°C, 5 min  38 cycles of:[ 94°C, 35 s; 60°C, 35 s; 72°C, 90 s]  72°C, 8 min | Wang et al., 2009 |
|  | *Glu-B3h* | CCACCACAACAAACATTAA | GTGGTGGTTCTATACAACGA | 94°C, 5 min  38 cycles of:[ 94°C, 35 s; 60°C, 35 s; 72°C, 90 s]  72°C, 8 min | Wang et al., 2009 |
|  | *Glu-B3i* | TATAGCTAGTGCAACCTACCAT | TGGTTGTTGCGGTATAATTT | 94°C, 5 min  38 cycles of:[ 94°C, 35 s; 58°C, 35 s; 72°C, 90 s]  72°C, 8 min | Wang et al., 2009 |
|  | *Glu-B3bef* | GCATCAACAACAAATAGTACTAGAA | GGCGGGTCACACATGACA | 94°C, 5 min  38 cycles of:[ 94°C, 35 s; 60°C, 35 s; 72°C, 90 s]  72°C, 8 min | Wang et al., 2009 |
| *Glu-D3* | *GluD3-21/22* | TTGGGCCTAATCGCTCGC | TAGTCTCCATCTGCGCAATT | 94°C for 5 min  38 cycles of: [94°C, 40 s; 60°C, 40 s; 72°C, 90 s]  72°C for 5 min | Zhao et al., 2007 |
|  | *GluD3-22* | CTCGTCTTTGCCCTCCTCA | CTAAACAACGGTGACCCAAT | 94°C for 5 min  38 cycles of: [94°C, 40 s; 60°C, 40s; 72°C, 60 s]  72°C for 5 min | Zhao et al., 2007 |
|  | *GluD3-23* | TCTGTACTTTGTGTGTGATCG | ACTGCTGCTGGAGGAATAG | 94°C for 5 min  38 cycles of: [94°C, 40 s; 59°C, 40 s; 72°C, 60 s]  72°C for 5 min | Zhao et al., 2007 |
|  | *GluD3-31* | ACAAGTGCCATTGCACAAATG | GATAGATGGATGAACAAATA | 94°C for 5 min  38 cycles of: [94°C, 45 s; 56°C, 45 s; 72°C, 80 s]  72°C for 5 min | Zhao et al., 2007 |
|  | *GluD3-32* | CAAGTGCCATTGCACAAATT | AATGATGGTTGTTGCGGTAT | 94°C for 5 min  38 cycles of: [94°C, 30 s; 59°C, 30 s; 72°C, 60 s]  72°C for 5 min | Zhao et al., 2007 |
|  | *GluD3-41* | AAGTAGTTAGCACCAATCCAT | CCTGTTGTTGTTGTTGTTGTT | 94°C for 5 min  38 cycles of: [94°C, 45 s; 59°C, 45 s; 72°C, 90 s]  72°C for 5 min | Zhao et al., 2007 |
|  | *GluD3-43* | GCATCAAAACCAAGCAAAAG | GGCTGAACAATAGGGATTTA | 94°C for 5 min  38 cycles of: [94°C, 30 s; 61°C, 30 s; 72°C, 60 s]  72°C for 5 min | Zhao et al., 2007 |
| *H22* | *Xgdm33* | GGCTCAATTCAACCGTTCTT | TACGTTCTGGTGGCTGCTC | 94°C , 5 min  35 cycles of: [94°C, 30s; 59°C, 30s; 72°C, 45s]  72°C, 5 min | Zhao et al., 2006  Standard SSR- PCR Program used in our laboratory |
